# *Pleurotus* spp. Mycelia Enriched in Magnesium and Zinc Salts as a Potential Functional Food

**DOI:** 10.3390/molecules26010162

**Published:** 2020-12-31

**Authors:** Anna Włodarczyk, Agata Krakowska, Katarzyna Sułkowska-Ziaja, Małgorzata Suchanek, Piotr Zięba, Włodzimierz Opoka, Bożena Muszyńska

**Affiliations:** 1 Department of Pharmaceutical Botany Jagiellonian, Faculty of Pharmacy, University Medical College, 9 Medyczna Street, 30-688 Kraków, Poland; annawlodarczyk1966@gmail.com (A.W.); katarzyna.sulkowska-ziaja@uj.edu.pl (K.S.-Z.); 2 Department of Inorganic and Analytical Chemistry, Faculty of Pharmacy, Jagiellonian University Medical College, 9 Medyczna Street, 30-688 Kraków, Poland; wlodzimierz.opoka@uj.edu.pl; 3 Department of Analytical Chemistry and Biochemistry, Faculty of Materials Science and Ceramics, AGH University of Science and Technology, Al. Mickiewicza 30, 30-059 Kraków, Poland; msuchanek@agh.edu.pl; 4 Department of Horticulture, Faculty of Biotechnology and Horticulture, University of Agriculture in Kraków, 29 Listopada 54, 31-425 Kraków, Poland; p.zieba90@gmail.com

**Keywords:** *Pleurotus* spp., antioxidative effect, bioelements, anions, phenolic compounds, indole compounds, digestive tract

## Abstract

Worldwide, mushrooms belonging to the *Pleurotus* spp. such as *P. citrinopileatus*, *P. djamor*, and *P. pulmonarius* are highly valued not only for their taste and aroma but also for their health-promoting properties. These species are rich in bioelements, vitamins, and above all, compounds that exhibit immunostimulatory activity. Therefore, in this study, we aimed to determine the effect of the supplementation of culture media using inorganic Mg and Zn salts. This is the first study to establish the bioavailability of the selected elements (Mg and Zn) and anions (Cl^−^, SO_4_^2−^) from the enriched biomass by means of the extraction of lyophilized mycelium into artificial digestive juices. The following salts were added to the liquid Oddoux medium: ZnSO_4_·7H_2_O, ZnCl_2_, MgSO_4_·7H_2_O or MgCl_2_·6H_2_O. The bioelements, anions and organic compounds in the obtained biomass were determined. The addition of Zn and Mg salts to the media increased the production of biomass by 30% and increased the bioaccumulation of the inorganic salts. Maintaining in vitro cultures under optimized and controlled conditions produced mycelium with a better composition and health properties than otherwise. Such enriched biomass may be classified as potential functional foods, aiding in overcoming deficiencies of elements and organic compounds with biological activity in humans.

## 1. Introduction

Edible mushrooms are valued by consumers not only for their taste and aroma but also for their health-promoting properties. Over 700 species of mushrooms are known to impart beneficial effects on human health. Mushrooms with medicinal properties have been utilized in traditional medicine [1,2,3]. Scientific publications since 2000 have confirmed the health-promoting properties of mushrooms. They exhibit anti-inflammatory, immunostimulatory, antidiabetic, antioxidative, hepatoprotective, anticancer, anti-atherosclerotic, antiviral, antibacterial, and antifungal properties [4,5,6]. Mushrooms are rich in vitamins, bioelements, phenolic compounds, indole compounds, carotenoids, and tocopherols [7,8,9].

Mushroom cultivation is one of the most dynamically developing branches of the contemporary food industry in many countries. China is the global leader in the production of mushrooms (90% of global production). *Lentinula edodes* (Shiitake) is the most popularly cultivated species—approximately two million tons are cultivated annually—but the global production of different *Pleurotus* spp. is almost at the same level [10,11].

In Asia, the use of mushrooms for food and medicines stems from centuries-old traditions, which have been documented more extensively than those in Europe. In Europe, the cultivation of *Pleurotus* spp. gained importance during World War II, when the health-promoting properties of mushrooms were discovered [12]. They constituted the basis for a diet that is rich in vital nutrients. Today, the most commonly cultivated species is *Pleurotus ostreatus*; however, other representatives of this genus, such as *P. eryngii*, *P. djamor*, and *P. citrinopileatus* are also gaining popularity among the consumers [13].

The fruiting bodies and biomass obtained from the in vitro cultures of *Pleurotus citrinopileatus*, *Pleurotus djamor*, and *Pleurotus pulmonarius* contain biologically active compounds. They exhibit antioxidant, antiviral, and antibacterial properties. Oyster mushrooms contain β-glucans which exhibit numerous clinically proven immunostimulatory properties. They are rich in exo- and endogenous amino acids, unsaturated fatty acids, vitamins, and macro- and micronutrients that are highly valued [14].

The unique capability of edible mushrooms, including *Pleurotus* spp., to accumulate elements has been the subject of numerous studies [8,14,15]. Moreover, the earliest reports on the bioaccumulation of certain metals in mushrooms were published nearly a century ago [16]. Initially, researchers focused on the assessment of the toxicity of mushrooms, resulting from the accumulation of heavy metals. Later, practical possibilities of mushroom application as natural diet components enriched with selected elements were recorded [17]. The absorption of elements by mycelium from in vitro cultures is strictly related to the availability of metals in the substrate [18,19,20]. Therefore, the culture medium needs to be appropriately modified to obtain mycelium from in vitro cultures that is characterized by a high content of cations and anions, as well as having biologically active organic compounds [21,22]. Bioelements, cations, and anions fulfill an important role in the growth and functioning of humans [23,24,25]. Their deficiency might lead to various ailments, which people attempt to minimize by consuming dietary supplements. To the best of our knowledge, there are no scientific data regarding the effect of supplementation of culture medium on the number of bioactive components in the mycelium of *P. citrinopileatus*, *P. djamor*, and *P. pulmonarius*, as well as their bioavailability in humans. Therefore, the aim of this study was to obtain the enriched biomass with inorganic salts biomass cultivated in in vitro conditions and in crops. In addition, we determined the effect of the supplementation of culture media on the accumulation of the given element in the mycelium, as well as the content of bioactive organic compounds. Furthermore, we tested the release of these components into the artificial digestive juices to determine their bioavailability.

## 2. Materials and Methods

### 2.1. Mushroom Materials

#### 2.1.1. Initial Mycelial Cultures 

In this study, mycelial cultures of three *Pleurotus* species were selected: *P. citrinopileatus* Singer, *P. djamor* (Rumph. ex Fr.) Boedijn, and *P. pulmonarius* (Fr.) Quel. Mém. Soc. Émul. Montbéliard, Sér. cultured on solid medium according to Oddoux (1957) [26] (deposit of the Department of Pharmaceutical Botany, Jagiellonian University Medical College). The biomass of in vitro cultures on the solid medium was passaged (at the amount of 0.1 g inoculum to Erlenmeyer flasks with 500 mL volume, filled with 250 mL of liquid medium). The established liquid cultures were described in our previous publications [8,14]. 

#### 2.1.2. Experimental Mycelial Cultures

**Figure 1:**
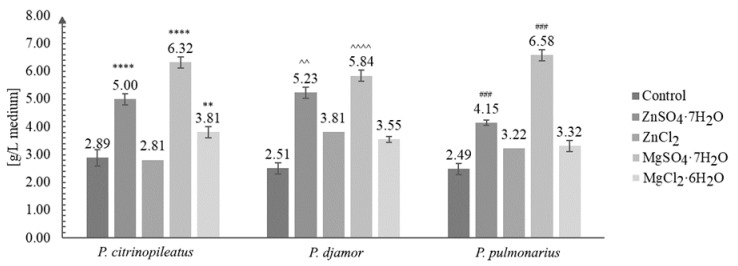
Biomass obtained after in vitro culture of *Pleurotus citrinopileatus*, *Pleurotus djamor*, and *Pleurotus pulmonarius* on the liquid Oddoux medium (g/L of medium) (one-way ANOVA with post-hoc Tukey’s test: ** *p* < 0.01 vs. *P. citrinopileatus* control; **** *p* < 0.0001 vs. *P. citrinopileatus* control; ^^ *p* < 0.01, ^^^^ *p* < 0.0001 vs *P. djamor* control; ### *p* < 0.001 vs. *P. pulmonarius* control).

Erlenmeyer flasks with 250 mL of basic Oddoux medium were supplemented by one of the following inorganic salts separately for each of *Pleurotus* species: ZnSO_4_·7H_2_O (100 mg/L of medium), ZnCl_2_ (40 mg/L of medium), MgSO_4_·7H_2_O (1980 mg/L of medium) or MgCl_2_ ·6H_2_O (3340 mg/L of medium) [27]. Each salt was added in three independent repetitions. After 21 days of growth, the mycelia from liquid cultures were separated (Figure 1) by filtration (using a Pyrex Buchner funnel, with Whatman^®^ qualitative filter paper, Grade 1, Merck) from the medium, washed several times by quadruple distilled water, frozen, and lyophilized (Freezone 4.5 lyophilizer, Labconco; temperature: −40 °C). After freeze-drying, mycelia were powdered in an agate mortar. The growth of mycelium was evaluated as a final biomass of the mycelium after cultivation in uniform volumes of liquid medium.

#### 2.1.3. Fruiting Bodies 

In the first stage, the biomass obtained from the in vitro cultures was used to obtain fruiting bodies of *Pleurotus* spp. at the cultivation laboratory of the Department of Horticulture of the University of Agriculture in Krakow. Sporocarps of *Pleurotus* spp. species were used in the experiment as a supplementary control, to compare it with mycelia from in vitro culture control in artificial digestive juices. After the cultivation, granular mycelium was obtained. In the next stage, the wheat kernels with suitable humidity were placed in polypropylene bags and were subjected to sterilization at 121 °C for 1.5 h and a pressure of 1 atm. After cooling, the medium was incorporated with mycelium obtained from the in vitro cultures of the selected *Pleurotus* spp. Next, the cultivation substrate was prepared by using a mixture of beech sawdust, ground wheat straw, and wheat bran (weight ratio of 6:1:3 and 1% of gardening gypsum). After a thorough homogenization of the substrate, it was moisturized to obtain a moisture level of 65% and was transferred to polypropylene bags with microfilter. The substrate (2.5 kg) was placed in each of the prepared bags and was subjected to sterilization (121 °C, for 1.5 h and at a pressure of 1 atm). The cooled bags were inoculated with 3% granular mycelium (75 ± 1 g per one 2.5 kg cube) obtained in the first stage, mixed with substrate in bags, and molded into cultivation cubes, which were incubated in the dark at 24 ± 1 °C until fully overgrown by mycelium. The cultivation cubes were placed in cultivation chambers, in which constant physicochemical conditions were maintained: 90 ± 3% humidity, 18 ± 2 °C, and photoperiod 12 h of light at 900 lx intensity, and 12 h without light. These conditions were previously optimized for the tested mushroom species. Fruiting bodies were collected after they reached the harvesting maturity. For the analysis, only uniform fruiting bodies with a typical appearance from the first batch were selected. The samples were lyophilized and homogenized to obtain a uniform powder.

### 2.2. Reagents

In this study, the following reagents were used for the mineralization of the lyophilized biomass and fruiting bodies: 65% HNO_3_ and 30% H_2_O_2_ Suprapure both from Merck (Darmstadt, Germany). Metal content standards for Mg(II) and Zn(II) with 1 g/L concentration were purchased from the District Measurements Office in Łódź (Poland). The liquid culture media were enriched with salts: ZnSO_4_·7H_2_O (Cat. No.: 204986) and ZnCl_2_ (Cat. No.: 229997) and MgSO_4_·7H_2_O (Cat. No.: 63138) and MgCl_2_·6H_2_O (Cat. No.: M2670), purchased from Sigma-Aldrich (Darmstadt, Germany).

MgCl_2_, NaCl and NaHCO_3_ were from PPH Golpharm (Kraków, Poland); pepsin and bile salts were from BTL (Łódź, Poland); CaCl_2_ was from Pharma Zentrale GmbH (Germany); pancreatic extract, HCl, KCl, HNO_3_ concentrated, Suprapur^®^, and KNO_3_, Suprapur^®^ were obtained from Merck (Darmstad, Germany); C_6_H_8_O_7_, KHCO_3_, Na_2_HPO_4_, K_2_HPO_4_, and NaOH was from the Polish Company of Chemistry (Gliwice, Poland); standards of indole compounds were from Sigma-Aldrich (St. Louis, MO, USA); all these compounds were of HPLC grade. Standards of phenylalanine and phenolic acids were from Sigma-Aldrich (St. Louis, MO, USA). The analytical grade methanol, acetic acid and ammonium acetate were purchased from Chempur, Gliwice, Poland. HPLC-grade methanol was purchased from Honeywell Riedel–de Haën, Seelze, Germany. Water (quadruple-distilled) with a conductivity of less than 1 µS/cm was obtained using an S2-97A2 distillation apparatus (ChemLand, Stargard Szczecin, Poland).

### 2.3. Preparation of Artificial Digestive Juices 

In this study, the artificial digestive juices (saliva, gastric and intestinal juice) were prepared as shown in Table 1. The ingredients were weighed on analytical scales with an accuracy of 0.1 mg. All the components were transferred into a 1000 mL flask and topped with quadruple distilled water.

### 2.4. Extraction of Metals and Organic Compounds into Artificial Digestive Juices

The powdered agate mortar samples were subjected to the action of artificial digestive juices to determine the content of metals (Zn and Mg) and organic compounds (phenolic compounds, indole compounds, and selected amino acids) released into the artificial digestive juices. We performed three independent repetitions for the extraction. Briefly, 0.5 g of sample was placed in a flat-bottomed flask. Subsequently, the biomass was moisturized with 3 mL of artificial saliva and 20 mL of artificial gastric juice. Then, the mixture was placed for 60 min in a Gastroel-2014 apparatus, which was constructed at the Chair of Inorganic and Analytical Chemistry at the Faculty of Pharmacy, Jagiellonian University Medical College, which enabled testing in conditions that were as close as possible to the physiological conditions of the organism (37 °C) [32]. After 1 h, the content of the flask was filtered through membrane filter paper (Ø 0.22 μm, Millex, Millipore Corporation, Burlingtone, MA, USA). To the filtrate, 20 mL of artificial gastric juice were added, and the digestion process continued for another 150 min in the Gastroel-2014 apparatus (Kraków, Poland). Subsequently, the mixture was filtered. In this way, the period of digestion with the artificial intestinal juice, which was approximately the physiological period in the human digestive system, was ensured. The content of elements and organic compounds were determined in the filtrates as described below. 

### 2.5. Analysis of Organic Compounds—Preparation of the Extract

Briefly, 3 g of powdered fruiting bodies and mycelia were extracted with methanol by ultrasound (49 kHz for 30 min; Sonic-2, Polsonic, Warsaw, Poland). The extraction was repeated in triplicate for each species from three independent samples (fruiting bodies and mycelia). The obtained extracts were combined (300 mL) and evaporated to dryness. Subsequently, the extracts were quantitatively dissolved in HPLC grade methanol and filtered using membrane filters. 

### 2.6. Analytical Tools Applied

#### 2.6.1. Analysis of Mg and Zn

**Figure 2:**
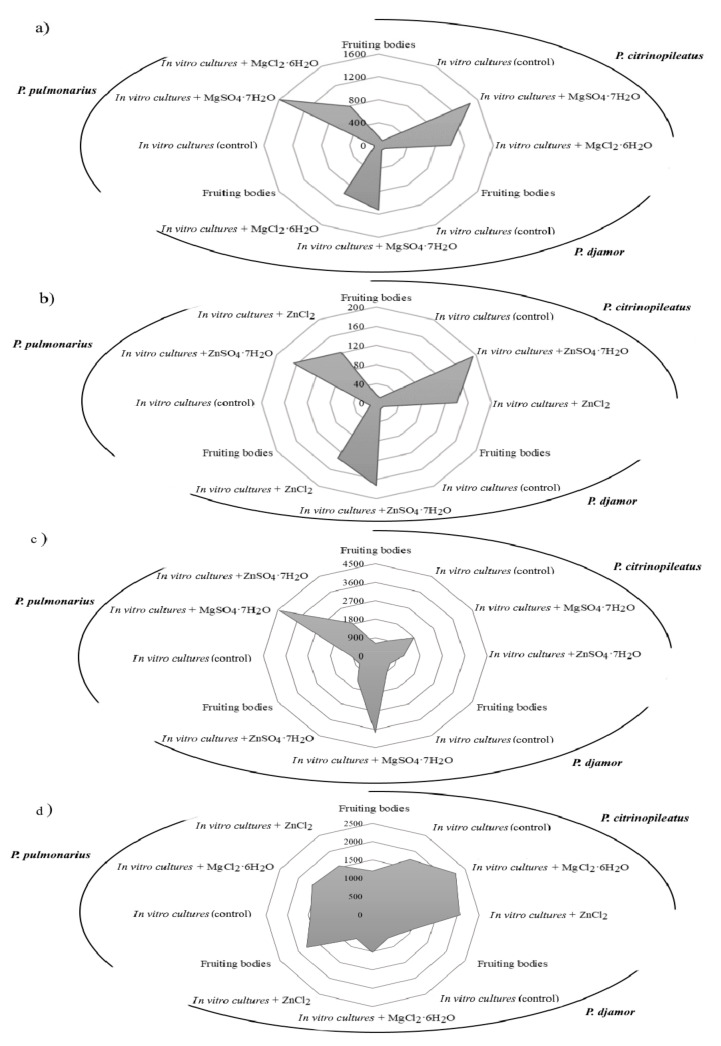
The concentration of (**a**) Mg and (**b**) Zn (mg/100 g d.w.) and the concentration of anions: (**c**) SO_4_^2−^ and (**d**) Cl^−^ in the fruiting bodies of *Pleurotus citrinopileatus*, *Pleurotus djamor*, and *Pleurotus Pulmonarius* after in vitro cultivation in media enriched with zinc (ZnSO_4_·7H_2_O and ZnCl_2_) and magnesium salts (MgSO_4_·7H_2_O and MgCl_2_·6H_2_O).

These samples were analyzed for the content of Zn and Mg. Briefly, three independent samples (0.2 g) were weighed from each of the lyophilized powdered fruiting bodies, and mycelia from in vitro cultures were transferred into Teflon vessels containing 2 mL of 30% H_2_O_2_ and 6 mL 65% HNO_3_. Then, the samples were subjected to wet mineralization in a closed system in a Magnum II mineralizer (ERTEC). The obtained mineralized solution was heated on a hotplate for 60 min at 120 °C to remove excess reagents. Subsequently, all the samples were quantitatively transferred flasks and topped to 10 mL with quadruple distilled water. To determine elements, flame atomic absorption spectrometry (FAAS) was used. For all measurements, the atomic absorption spectrometer by Thermo Scientific (Model iCE 3500, Cambridge, UK) was used. Three samples of each fruiting bodies and mycelia were analyzed in three repetitions, and the results are presented as mean ± standard deviation (SD) for each sample (based on 3 repetitions) (Figure 2a,b and Table 2).

#### 2.6.2. Determination of Chloride and Sulfate Ions

Cl^−^ and SO_4_^2−^ were determined using spectrophotometry with Spectroquant Nova 60 spectrophotometer (Merck KGaA, Darmstadt, Germany). We used validated tests to determine SO_4_^2−^ (Cat. No.: 101812, Merck KGaA, Darmstadt, Germany) and Cl^−^ (Cat. No.: 114897, Merck KGaA, Darmstadt, Germany). Spectrophotometric determinations were performed in quartz cuvettes. Samples from intestinal juices were filtered by using a 0.45 μm membrane (Merck KGaA, Darmstadt, Germany). The results of the determination of the content of Cl^−^ and SO_4_^2−^ anions are presented as mean values from three independent measurements and are summarized in Table 2. In each of the discussed cases, the independent result was corrected for the amount of background (the content of individual anions presents in the control sample − digestive juice solution).

#### 2.6.3. Determination of Phenylalanine and Phenolic Acids

In order to conduct the analysis of phenylalanine and phenolic acids, reverse phase high-performance liquid chromatography (RP-HPLC) with a diode array detector (DAD) was used. For the analysis, HPLC VWR Merck Hitachi apparatus (Tokyo, Japan) was used with an autosampler (L–2200), pump (L–2130), RP–18e LiChrospher column (4 mm × 250 mm, 5 µm) maintained at 25 °C, and thermostat (L–2350). DAD (L–2455), operating in the wavelength range of 200–400 nm. The mobile phase was prepared as follows: solvent A: methanol/0.5% acetic acid 1:4 (*v/v*) and solvent B: methanol. Gradient was set as follows: 100:0 time 0–25 min; 70:30 time 35 min; 50:50 time 45 min; 0:100 time 50–55 min; 100:0 time 57–67 min. The comparison of UV spectra and retention times relative to the standard compounds enabled the identification of phenolic compounds. The quantitative analysis was performed using a calibration curve. The results of phenolic content determination in fruiting bodies, mycelium from in vitro cultures, and digestive juices were expressed in mg/100 g dry weight (d.w.).

#### 2.6.4. Analysis of Indole Compounds

The extracts were analyzed for the content of indole compounds using the RP-HPLC method with UV detection. The prepared extracts were quantitatively dissolved in 1.5 mL of the solvent mixture and the components were separated via RP-HPLC method (Hitachi RP-HPLC with UV detection, Merck, Tokyo, Japan) equipped with an L-7100 type pump. The Purospher^®^ RP–18 column (4 mm × 200 mm, 5 µm) was maintained at 25 °C and was equipped with a UV detector (λ = 280 nm). The applied liquid phase consisted of a mixture of methanol/water/ammonium acetate (15:14:1 *v/v*). The flow rate was established at 1 mL/min. Indole compounds were quantitatively analyzed with the help of a calibration curve and with the assumption of linearity of the size of the area tested under the peak relative to the concentration of the standard used. Table 3 shows the results obtained, and the results are expressed in mg/100 g d.w.

N = 9; n.d.—not detected; *—lower than the limit of detection; values followed by a different letter (a, b, c, d, e, f) within the same row are significantly different (*p* < 0.05).

#### 2.6.5. Determination of the Total Phenol Content

Total phenol content was determined using the Folin–Ciocalteu method. Briefly, 0.1 mL of methanolic extract (prepared as described in the previous subsection) was added to Folin–Ciocalteu reagent (Merck KGaA, Darmstadt, Germany). In the case of digestive juices, 0.1 mL of the juice was added without dissolution. The reduction of molybdenum(VI) to molybdenum(V) by phenolic compounds resulted in a blue-colored product which showed maximum absorption at 745–750 nm. The intensity of the color formed was measured using the UV-VIS Helios Beta spectrophotometer (Thermo Fisher Scientific Inc., Waltham, MA, USA). The total phenolic content was calculated based on a calibration curve and was expressed in mg/100 g of d.w., converted into gallic acid equivalents. For the digestive juices, the phenols compounds were converted into mg/mL of gallic acid.

### 2.7. Determination of Antioxidant Activity Using the DPPH Method

The antioxidant activity was determined using 1,1-diphenyl-2-picrylhydrazyl (DPPH) radical (Sigma-Aldrich, St. Louis, MO, USA). Briefly, 2 g of fruiting bodies and mycelium from in vitro cultures was extracted with 8 mL of methanol. From this methanolic extract, 0.1 mL was added to 4.9 mL of 0.1 mM DPPH solution dissolved in 100% methanol. In the case of digestive juices, 0.1 mL of the artificial juice was added without dissolution. The reaction mixtures were incubated for 20 min in darkness at 22 °C. Subsequently, the absorbance was measured at 517 nm by using UV–VIS Helios Beta spectrophotometer (Thermo Fisher Scientific Inc., Waltham, MA, USA). The DPPH radical reduction was calculated using the following equation (1):

(1) $$AA(\%)=\left[\frac{\left(A_{0}-A_{1}\right)}{A_{0}}\right]\times 100$$

where *AA* is the antioxidant activity expressed in %, *A*_0_ is the absorbance of blank/zero sample, and *A*_1_ is the absorbance of the tested concentration.

### 2.8. Statistical Analysis

All the test samples were analyzed in three isolates of three repetitions of each species, and the results are expressed as mean ± SD. Tukey’s test was applied to elaborate on the results of the determinations of elements. Statistically significant differences in mycelium from in vitro culture growth in the analyzed *Pleurotus* spp. species were analyzed using one-way ANOVA with post-hoc Tukey’s test. The statistical significance level was set at *p* < 0.05 (GraphPad InStat). Data were statistically analyzed using the Statgraphics Centurion XVIII software. We analyzed the data using the cluster analysis (CA) method and principal component analysis (PCA). The use of these methods allowed for a reduction in input data and enabled the identification of object groups that were correlated.

## 3. Results and Discussion

In this study, our results demonstrated the positive effect of the addition of inorganic salts (ZnSO_4_·7H_2_O and ZnCl_2_ and MgSO_4_·7H_2_O and MgCl_2_·6H_2_O) on the growth of the mycelium of *P. citrinopileatus*, *P. djamor*, and *P. pulmonarius* under in vitro conditions (Figure 1). For comparison, we also maintained control cultures (mycelium without the addition of the salts).

### 3.1. Biomass Growth Analysis

Figure 1 shows the growth of the biomass under in vitro conditions.

The increase in biomass after in vitro culture in media enriched with inorganic salts was greatly enhanced compared with that of cultures grown without the addition of inorganic salts. The greatest amount of growth, three-fold (to be exact 2.64-fold on average), was obtained for *P. pulmonarius* grown on the medium enriched with MgSO_4_·7H_2_O salt—6.58 g/L of medium (control: 2.89 g/L of medium). The least amount of growth was obtained for the same species (*P*. *pulmonarius*) cultivated on the same medium without the addition of salt. The most efficient growth was obtained for the cultures grown on the media enriched with ZnSO_4_·7H_2_O and MgSO_4_·7H_2_O. A previous study [33] reported the highest yield of biomass (5.49 g/L) for *Pleurotus sajor-caju*. Other studies reported the yield between 9.7–22.8 g dry mycelium/L of medium, when different concentrations of glucose were added to the medium [34,35,36]. The results obtained in this study suggest that the increased quantity of biomass obtained is due to the addition magnesium sulfate. This might be of key importance for industries where the commercial production of dietary supplements or drugs requires a greater quantity of the initial raw material available.

### 3.2. Analysis of Metals and Anions Content 

In the first stage of the study, we determined the level of selected bioelements (Mg and Zn) and anions (Cl^−^ and SO_4_^2−^) present in the mycelium obtained from the in vitro cultivation on the media enriched with the addition of inorganic salts of zinc (ZnSO_4_·7H_2_O and ZnCl_2_) and of magnesium (MgSO_4_·7H_2_O and MgCl_2_·6H_2_O) (Figure 2a,b) and in the fruiting bodies of *P. citrinopileatus*, *P. djamor*, and *P. pulmonarius*.

In this study, the addition of inorganic salts to mycelial cultures resulted in the increased accumulation of the elements (Mg and Zn) in the obtained biomass. The lowest amount of Mg and Zn was detected in the mycelium from the control cultures (Mg: 101.1 mg/100 g d.w.—*P. citrinopileatus*, 84.6 mg/100 g d.w.—*P. djamor*, and 61.8 mg/100 g d.w.—*P. pulmonarius*; Zn: 12.4 mg/100 g d.w.—*P. citrinopileatus*, 15.1 mg/100 g d.w.—*P. djamor*, and 18.6 mg/100 g d.w.—*P. pulmonarius*). The highest level of Mg and Zn was detected in the mycelia grown on the media enriched with MgSO_4_·7H_2_O and ZnSO_4_·7H_2_O salts. The highest content of Mg was found in the mycelium of *P. pulmonarius* (1593 mg/100 g d.w.) grown on the medium enriched with MgSO_4_·7H_2_O, whereas the highest content of Zn was found in the mycelium of *P. citrinopileatus* (193.4 mg/100 g d.w.) grown on the medium enriched with ZnSO_4_·7H_2_O. Enriching the culture medium with chloride salts (MgCl_2_·6H_2_O, ZnCl_2_) also increased the accumulation of metals in the obtained biomass but was less efficiently: Mg: 1011.4 mg/100 g d.w.—*P. citrinopileatus*, 973.4 mg/100 g d.w.—*P. djamor*, and 792.6 mg/100 g d.w.—*P. pulmonarius*; Zn: 140.6 mg/100 g d.w.—*P. citrinopileatus*, 132.5 mg/100 g d.w.—*P. djamor*, and 121.9 mg/100 g d.w.—*P. pulmonarius*). Previous studies have focused on the accumulation of Zn in the fruiting bodies of *Pleurotus* spp. and not in the mycelium obtained via biotechnological methods. Bioelement concentrations in fruiting bodies are markedly lower [36] and by means of comparison, *P. ostreatus* and *P. eryngii* fruiting bodies were obtained supplemented with zinc, containing 6 mg Zn per 100 g d.w. Poursaeid et al. [37] obtained 189 mg Zn from 100 g d.w. of *P. florida* mycelium supplemented with 200 mg Zn/L medium. In another experimental work, Krakowska et al. [21] using in vitro culture supplementation of another species of mushroom—*A. bisporus*—in inorganic zinc salts ZnSO_4_·7H_2_O (200 mg/L medium) and magnesium MgSO_4_·7H_2_O (2000 mg/L medium) also achieved higher adsorption efficiency: Mg—2696 mg/100 g d.w., Zn—446.2 mg/100 g d.w. A similar effect was observed in other experiments [38] where in vitro cultures *L. edodes* were conducted on substrates with the addition of ZnSO_4_ (174.47 mg/L medium) and C_8_H_12_N_2_O_8_Zn—zinc hydrogen aspartate (200 mg/L medium)—in both cases, the addition of salt increased the amount of subsidized metal in the obtained biomass.

In this study, we obtained only 15.1 g of Zn from 100 g d.w. mycelium. So far, there have been no studies conducted on the supplementation of media with Mg for the growth of *Pleurotus* spp. mycelium. Our results suggest that mycelium is a good and a natural carrier of bioelements, and its usage as a dietary supplement for Mg and Zn might prove to be beneficial. The accumulation of Mg and Zn is considerably more pronounced in the mycelium than that in the fruiting bodies. Next, we studied the bioavailability of the abovementioned elements in humans from the biomass. To this end, we measured the amount of Mg and Zn extracted into the artificial digestive juices from the fruiting bodies and the lyophilized biomass obtained from the liquid medium, as well as from the biomass obtained on the medium enriched with Zn and Mg. According to the results, the elements (Mg and Zn) not only were utilized by the mycelia of *Pleurotus* spp. but were also released from the mycelia into the artificial digestive juices. Table 2 presents the results of the analysis of Mg and Zn content extracted into the artificial gastric juices from fruiting bodies and lyophilized biomass.

According to our results, the amount of the extracted metal (Mg and Zn) depends on the material from which the extraction was conducted (e.g., fruiting bodies, control mycelium, and mycelium enriched with elements) and on the type of environment from which the extraction was conducted (gastric or intestinal juice). We detected greater quantities of Zn and Mg in the digestive juice samples of biomass, which was grown on the medium enriched with ZnSO_4_·7H_2_O and MgSO_4_·7H_2_O. Furthermore, both Mg and Zn were more efficiently released into the gastric juice than that into the intestinal juice (Table 2).

In the gastric juice, the highest amount of Zn was extracted from *P. citrinopileatus* mycelium (166.3 mg/100 g d.w.), which was grown on the medium enriched with ZnSO_4_·7H_2_O, and the lowest amount of Zn was extracted from the mycelium grown on the medium enriched with ZnCl_2_ (111.3 mg/100 g d.w.). The lowest amount of Zn released into the gastric juice (98.8 mg/100g d.w.) was obtained for *P. pulmonarius*, and the quantity of Zn increased in the mycelium grown on the medium enriched with ZnCl_2_. In the case of intestinal juices, Zn was best released from the biomass obtained from *P. pulmonarius* (22.3 mg/100 g d.w.), which was grown the medium enriched with ZnSO_4_·7H_2_O. The lowest amount was obtained from in vitro cultures of *P. citrinopileatus* (6.4 mg/100 g d.w.), which were maintained on the medium enriched with ZnCl_2_ salts. It should be noted that the quantities of Zn released into the artificial digestive juices of the *Pleurotus* species were much higher than those which Muszyńska et al. [38] obtained for the species *L. edodes* (gastric juice—32.5 mg/100 g d.w. and intestinal juice—1.45 mg/100 g d.w.), research also carried out on a substrate enriched with inorganic zinc salt—ZnSO_4_. According to our results, *P. pulmonarius* released the highest quantity of Mg into the gastric juice (1280 mg/100 g d.w.), which was grown on medium enriched with MgSO_4_·7H_2_O. The quantity of Mg released into the artificial digestive juices was higher when the medium was enriched with MgSO_4_·7H_2_O than that of MgCl_2_·6H_2_O, which was 526.9 mg/100 g d.w. In the case of intestinal juice, the quantity of Mg extracted was considerably low. *P. djamor* grown on the medium enriched with MgSO_4_·7H_2_O released 156 mg/100 g d.w. Mg. In the case of *P. pulmonarius* mycelium grown on a medium enriched with MgCl_2_·6H_2_O salts, we obtained a low amount of Mg (132 mg/100 g d.w.). *P. pulmonarius* grown on the medium enriched with MgCl_2_·6H_2_O released the lowest amount of Mg (77 mg/100 g d.w.). As far as we know, for the first time, the content of SO_4_^2−^ and Cl^−^ ions were analyzed (Figure 2c,d). In the case of fruiting bodies of *Pleurotus* spp., SO_4_^2−^ ions were detected in similar quantities for: *P. citrinopileatus* (624 mg/100 g d.w.), *P. djamor* (692 mg/100 g d.w.), and *P. pulmonarius* (725 mg/100 g d.w.). The concentration of Cl^−^ ions in the fruiting bodies were substantially higher (*P. citrinopileatus*—1200 mg/100 g d.w., *P. djamor*—910 mg/100 g d.w., and *P. pulmonarius*—1777 mg/100 g d.w.). Higher levels of SO_4_^2−^ ions were determined in all the analyzed cases. Furthermore, MgSO_4_·7H_2_O provided more SO_4_^2−^ ions (*P. citrinopileatus*—1783 mg/100 g d.w., *P. djamor*—3750 mg/100 g d.w., and *P. pulmonarius*—4433 mg/100 g d.w.). However, the level of SO_4_^2−^ ions available from ZnSO_4_·7H_2_O was less (*P. citrinopileatus*—1142 mg/100 g d.w., *P. djamor*—1393 mg/100 g d.w., and *P. pulmonarius*—1862 mg/100 g d.w.) (Figure 2c). Similar results were obtained for Cl^−^ ions. In all the cases, we found that the addition of salts with Cl^−^ ions increased their level in the biomass. The greatest effect was recorded for Mg salts (*P. citrinopileatus*—2265 mg/100 g d.w., *P. djamor*—1020 mg/100 g d.w., and *P. pulmonarius*—1625 mg/100 g d.w.) (Figure 2d). However, the Cl^−^ and SO_4_^2−^ ions were extracted into the gastric juice more efficiently (Table 2). *P. citrinopileatus* grown on the media enriched with ZnCl_2_ salts exhibited the most efficient release of Cl^−^ ions—1067 mg/100 g d.w. However, the lowest amount of Cl^−^ ions was extracted in gastric juice of samples of *P. djamor* fruiting bodies—200 mg/100 g d.w. Furthermore, in the case of SO_4_^2−^ ions, the lowest level was recorded for *P. djamor* fruiting bodies—226 mg/100 g d.w., whereas the highest level was recorded for *P. pulmonarius* grown on the medium enriched with MgSO_4_·7H_2_O—3636 mg/100 g d.w.

According to our results, Mg, Zn, and Cl^−^ and SO_4_^2−^ ions were effectively released into the artificial gastric juices, making them bioavailable to humans. The daily requirement of Mg is about 300–420 mg, whereas that of Zn is about 2–12 mg. Regarding this, the daily requirement of Zn and 60% that of Mg can be easily met by the consumption of mycelium from in vitro cultures grown on enriched media. Moreover, the daily requirement of Cl^−^ (1850–2300 mg) and SO_4_^2−^ (about 460 mg) anions [27] can be partially met by the inclusion of mycelium from in vitro cultures enriched with the aforementioned inorganic salts, which might be considered as a functional food.

### 3.3. Determination of Organic Compounds

**Figure 3:**
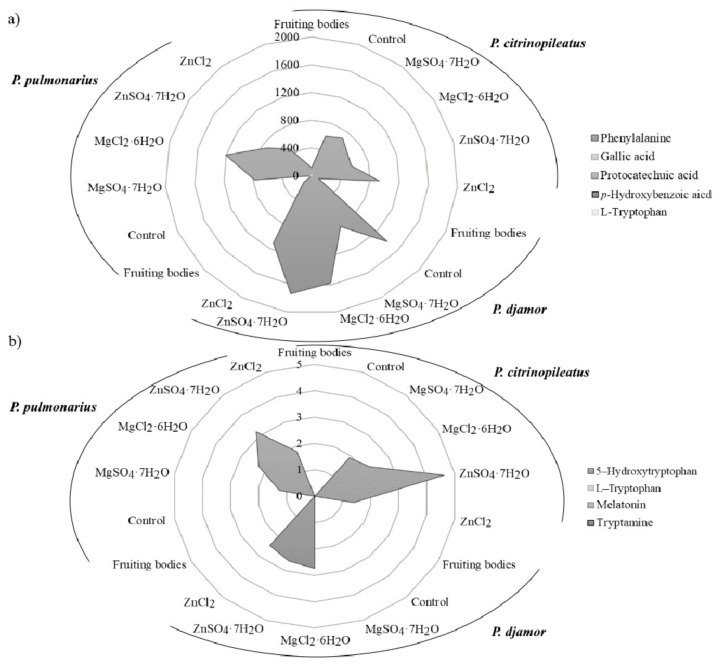
The content of organic compounds: (**a**) phenols and (**b**) indoles (mg/100 g d.w.) in the fruiting bodies of *Pleurotus citrinopileatus*, *Pleurotus djamor*, and *Pleurotus pulmonarius*, from in vitro cultures (control), and in vitro cultures enriched with zinc (ZnSO_4_·7H_2_O and ZnCl_2_) and magnesium (MgSO_4_·7H_2_O and MgCl_2_·6H_2_O).

The content of phenylalanine, phenolic compounds and indole compounds was determined in fruiting bodies and mycelia derived from in vitro cultures. For the first time, analysis of in vitro cultures with and without enrichment with inorganic salts also determined their content after the extraction with artificial digestive juices (Figure 3a,b).

The results confirmed the presence of phenylalanine in all of the analyzed samples, with the exception of *P. pulmonarius* mycelium (Figure 3a). The highest level of phenylalanine was detected in *P. djamor* (1405.6 mg/100 g d.w.) independently of the type of salt added. Furthermore, the highest level of phenylalanine was determined in the sample with the addition of ZnSO_4_·7H_2_O (1729.6 mg/100 g d.w.). The determined level of phenylalanine was more than 20 times higher than that reported in vegetables [39]. In the case of phenolic compounds, the mycelia from in vitro cultures of *Pleurotus* spp. contained gallic acid, protocatechuic acid, and *p*-hydroxybenzoic acid. The highest level of protocatechuic acid (1.48 mg/100 g d.w.—*P. djamor*) was found in cultures enriched with chloride salts (MgCl_2_·6H_2_O and ZnCl_2_). Among the indole compounds presented in the analyzed samples 5-hydroxy-L-tryptophan was dominated quantitatively (Figure 3b). The highest concentration was determined in the biomass of *P. citrinopileatus* from the medium containing ZnSO_4_·7H_2_O salt (4.62 mg/100 g d.w.). Melatonin was determined in the biomass of *P. citrinopileatus* grown on the medium with MgCl_2_·6H_2_O salt (0.02 mg/100 g d.w.). Tryptamine was detected in all of the investigated mycelia from in vitro cultures of *Pleurotus* spp. that were grown on the media containing chloride salts (MgCl_2_·6H_2_O and ZnCl_2_) (0.04 mg/100 g d.w.). The level of phenolic and indole compounds was similar to that determined previously in another species of edible mushrooms [2,40]. According to the results, the amount of phenylalanine, phenolic and indole compounds extracted into the artificial digestive juices showed results similar to that of metals. The organic compounds were released in the greatest amounts into the artificial gastric juice (Table 3). Phenylalanine was more efficiently released into the gastric juice than that of other organic compounds. Its highest levels were detected in the extract obtained from the biomass of *P. djamor* (721.64 mg/100 g d.w.) propagated on the culture medium enriched with MgSO_4_·7H_2_O salt. However, its lowest quantity was detected in the biomass of *P. pulmonarius* (35.72 mg/100 g d.w.) grown on the medium enriched with chloride salts (MgCl_2_·6H_2_O and ZnCl_2_). It should be emphasized that in each of the discussed cases, the addition of both SO_4_^2−^ and Cl^−^ salts to the media resulted in the increased content of phenylalanine in the obtained biomass. Higher quantities of the bioavailable phenylalanine meant enhanced antioxidative and health-promoting properties of the mushrooms. In the case of the analysis of gallic acid content, its greatest levels were obtained from the biomass grown on the medium enriched with sulfate salts (MgSO_4_·7H_2_O and ZnSO_4_·7H_2_O): *P. citrinopileatus*—2.43 mg/100 g d.w., *P. djamor*—2.10 mg/100 g d.w., and *P. pulmonarius*—0.15 mg/100 g d.w. However, *P. citrinopileatus* released the greatest amounts of indole compounds into the artificial digestive juices. In addition, in this case, the most efficient effect of sulfate salts on the amount of indole compounds extracted to digestive juices was determined, in particular on the content of 5-hydroxy-L-tryptophan: *P. citrinopileatus*—1.06 mg/100 g d.w., *P. djamor*—0.83 mg/100 g d.w., *P. pulmonarius*—0.93 mg/100 g d.w. Our previous research into the release of chemicals into gastric juices has shown the great potential of *P. ostreatus* fruiting bodies. High levels of phenolic acid and indole release were found, which underlines the nutritional value of this species [2,14].

### 3.4. Chemometric Analysis of Organic Compounds

**Figure 4:**
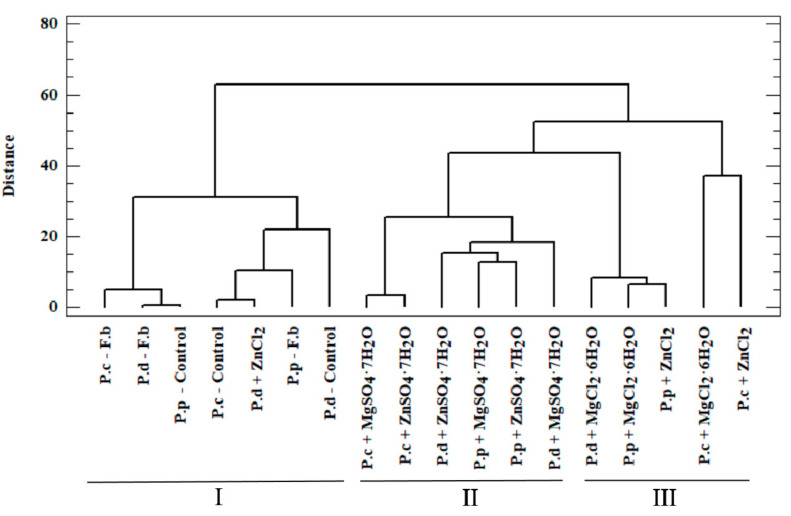
Dendrogram presenting similarity between the analyzed objects (Euclidean, City block algorithm) of added salts in liquid medium for the growth of mycelium from in vitro cultures of P.c—*Pleurotus citrinopileatus*, P.d—*Pleurotus djamor*, and P.p—*Pleurotus pulmonarius*, and Control—*in vitro* cultures without addition of salts. F.b—fruiting bodies.

To determine the actual bioavailability of the organic compounds contained in the biomass, we performed an in-depth analysis of the results. To this end, we performed a chemometric analysis based on two methods: cluster analysis (CA) and principal component analysis (PCA). In the case of CA, the test objects comprised *P. citrinopileatus*, *P. djamor*, and *P. pulmonarius* grown on the liquid media enriched with the addition of MgSO_4_·7H_2_O, ZnSO_4_·7H_2_O, MgCl_2_·6H_2_O, and ZnCl_2_. The analyzed objects were described using organic compounds as the parameters. The high variability in the data obtained justified the use of chemometric analysis in this study. Our analysis enabled us to obtain information on the correlations between the analyzed objects (mycelium from in vitro cultures and mushrooms of *Pleurotus* spp.) and the content of organic compounds measured in these objects (phenylalanine, phenolic acids, and indole compounds). Furthermore, our analysis enabled us to determine the effect of culture media on the growth of biomass enriched with bioelements and organic compounds, which is directly linked to the actual assessment of the bioavailability of these compounds for humans. In the case of CA, the substantial similarity of the objects (investigated mushroom species) was shown by their relatively close distribution in the multidimensional space. A dendrogram is a graphic result of the applied analysis (Figure 4). The dendrogram marks object clusters characterized by significant similarity. The axes presented in the Figure 4 ordinates and abscissae are not equal to the axes in the Cartesian coordinate system [41]. The analyzed objects were marked on axis *x*, whereas on axis *y*, the distance in between objects was marked, which were calculated using Ward’s agglomeration method. Based on CA, three clusters were distinguished, which are characterized by a similar variability. 

Within the first (I) cluster, the following objects are distinguished—fruiting bodies and in vitro cultures of the three analyzed *Pleurotus* species propagated without enriching the culture medium. The second (II) cluster consists of in vitro cultures obtained on the medium enriched with sulfate salts (MgSO_4_·7H_2_O and ZnSO_4_·7H_2_O), whereas the third (III) cluster is represented by in vitro cultures maintained on the medium enriched with chloride salts (MgCl_2_·6H_2_O and ZnCl_2_). Objects belonging to one group are similar within the analyzed clusters. This similarity is not only because of the similar structure of mushrooms but also the development of their absorption area [41]. The similarity within the analyzed clusters is also valuable information that medium modification also affects the course of bioelements and organic compound accumulation. It also shows the dependency on the type of additive used (sulfate or chloride salts) in the culture medium. The highest correlation was observed for cluster (I), which is indicated by the shortest branches of the dendrogram [42]. Unambiguous interpretation of such a wide set of analyzed objects was considerably limited. Therefore, PCA was applied as a complementary method in this study. This method helps to reduce the quantity of the analyzed data to the minimum, thereby enabling the explanation of the correlations contained therein. To this end, we defined the correlated factors, which are known as principal components. Based on PCA, it was determined that 73.3% of the variables in the analyzed input dataset can be explained by three principal components: PC1, PC_2_, and PC3. Thus, only the three principal components were taken into account for the next stage. The analyzed components corresponded to a linear combination of input variables multiplied by the loadings assigned to them (Table 4). 

**Figure 5:**
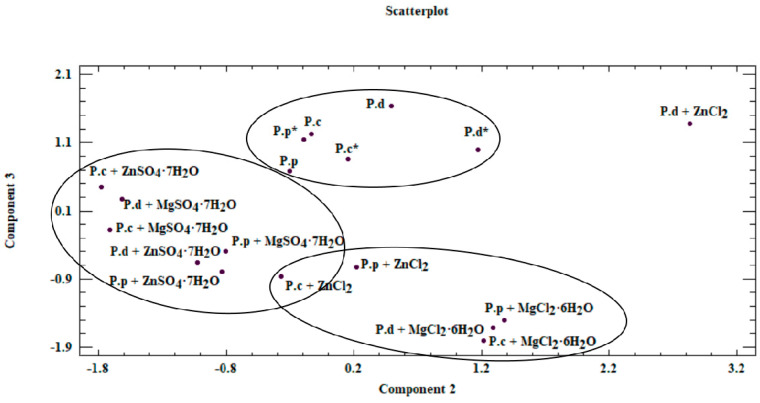
Scatterplot—graph presenting similarities between analyzed mycelium from in vitro cultures and fruiting bodies of *Pleurotus* species obtained on different culture media (P.c—*P. citrinopileatus* fruiting bodies, P.d—*P. djamor* fruiting bodies, P.p—*P. pulmonarius* fruiting bodies, *—mycelium obtained on a medium without the addition of inorganic salt).

Based on results of the PCA, the first component (PC1) was most significantly affected by the value measured for phenylalanine. This is associated with the assigned loading level, simultaneously indicating the correlation with the input variable. PC2 and PC3 components were analyzed analogously. Reduction in the analyzed area to three principal components enabled presenting the results on a two-dimensional plane (Figure 5).

**Figure 6:**
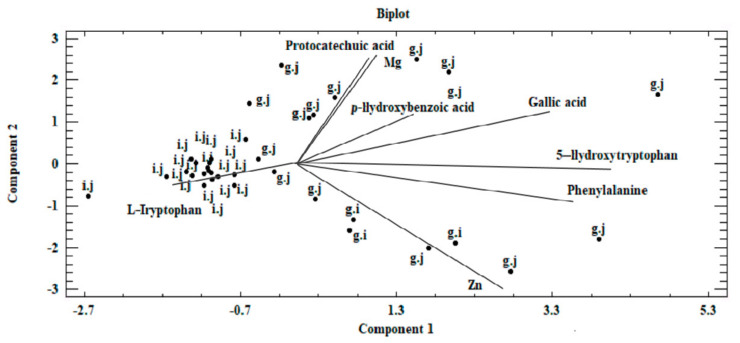
Biplot—graph on a two-dimensional plane, presenting the existing correlation in the analyzed dataset between components marked in the biomass obtained on liquid media enriched with inorganic salts for in vitro cultures of *Pleurotus citrinopileatus*, *Pleurotus djamor*, and *Pleurotus pulmonarius* and their extraction into artificial digestive juices (g.j—gastricjuice, i.j—intestinal juice).

Our analysis revealed that a correlation exists between the analyzed objects (Figure 5 and Figure 6). This similarity is not only derived from the ability of mycelium from in vitro cultures to accumulate bioactive substances, which in turn is affected by the modification of medium composition, as illustrated by the presence of three clusters in Figure 5. The first of these clusters consists of fruiting bodies and in vitro cultures grown without the addition of inorganic salts, whereas the two remaining clusters are in vitro cultures propagated on a liquid medium with the addition of sulfate and chloride salts. Furthermore, the similarity between the analyzed objects is affected by the efficiency in which the compounds present in the mushrooms are extracted to digestive juices. The discussed factors may stem from the similar structure of individual mushroom species (classified in one genus, *Pleurotus*) as a consequence of which similar release mechanisms of metals and organic compounds to artificial digestive juices occur, as presented in the biplot (Figure 6). Figure 6 was developed on the basis of the three principal components obtained (PC1, PC2, and PC3) and presents the course of the variability of the discussed metals and organic compounds relative to the site where their extraction to the digestive tract occurred. Based on this, it was shown that both metals and organic compounds were released into the artificial digestive juices from all the tested objects (mushroom species). In turn, the amount of released substances was associated with their extraction site (gastric and intestinal juice) and in both discussed cases, analysis of metals and organic compounds showed higher efficacy for the extraction in gastric juice. This trend further confirms the direction of the branches on the two-dimensional biplot graph. 

The planning of obtainment of natural diet components (mycelia) with the specified quantitative and qualitative composition is important information. In humans, the highest degree of absorption occurs in the intestine. Therefore, our analysis shows that the biomass obtained in this study, despite supplementing our daily requirement for the investigated bioelements, can help to supplement deficiencies of both metals and organic compounds, but may not constitute their sole resource for the system. In the final phase of the study, the antioxidative properties of the tested species were determined (Table 5). Analysis of antioxidant activity via the DPPH method showed that the addition of salts to media had a negative effect on the antioxidant properties of the mushroom material. The highest activity was demonstrated for *P. citrinopileatus* fruiting bodies, whereas the addition of MgSO_4_·7H_2_O resulted in a considerable reduction in the antioxidant activity of *P. pulmonarius* mycelium. However, the lowest activity was observed for the *P. pulmonarius* mycelium extract with the addition of ZnCl_2_. The addition of all salts to mycelium had a negative effect on the content of phenolic compounds; the highest content was found for *P. citrinopileatus* fruiting bodies and *P. pulmonarius* mycelium. Analysis of digestive juices revealed that intestinal juice contains trace amounts of phenolic compounds, and the amount of antioxidant activity was also minor (around 10%). This amount of activity was only recorded for the mycelium and fruiting bodies of *P. djamor*—supplementation of mycelium of this species reduced the antioxidant activity. Analysis of gastric juices showed increased antioxidative activity—reaching 43% for *P. pulmonarius*. In addition, higher content of phenolic compounds was found in gastric juice than that of intestinal juice.

Our study showed a correlation between enriching culture medium with metals for in vitro propagation, their accumulation in the mycelium, and their release from mycelium to digestive juices. We further confirmed the effect of increasing the content of organic compounds in the obtained biomass. The high content of metals and organic compounds depends on the conditions in which the fungal material is grown, thereby enabling us to obtain fruiting bodies and mycelium from in vitro cultures with controlled qualitative composition. This study adds to the application of such material as a valuable dietary source and as a functional food.

## 4. Conclusions

In this study, we conducted experiments on the accumulation and release of selected bioelements (Mg and Zn), anions (Cl^−^ and SO_4_^2−^) and bioactive organic compounds by mycelia of selected *Pleurotus* spp. The bioavailability of elements was studied based on their release into the artificial digestive juices, which provided new information on the benefits of consuming the studied mushrooms. The results show that mycelium from in vitro cultures of *P. citrinopileatus*, *P. djamor*, and *P. pulmonarius* accumulates elements and anions from the culture medium, and they release them into artificial digestive juices efficiently. The medium requires appropriate supplementation with metal salts, which results in increased growth of the mycelium from in vitro cultures and the degree of accumulation of elements. It also increases the amount of total phenolic content that demonstrates antioxidant activity. It has been proven that mycelium from in vitro cultures obtained Mg and Cl^−^ from a liquid medium enriched in salt content, and that these metals and anions were successively release in an in vitro digestive system. Thus, the enrichment of mycelium from in vitro cultures of three *Pleurotus* spp. with bioelements seems valid and has been proven to be a functional food for humans. 

## Figures and Tables

**Table 1:** Composition of artificial digestive juices [28,29,30,31].

Compounds	Sample Weight (g)
**Saliva (pH = 6.7)**	
KH_2_PO_4_ (25 mmol/L)	0.34
Na_2_HPO_4_ (24 mmol/L)	0.34
KHCO_3_ (150 mmol/L)	1.50
MgCl_2_ (1.5 mmol/L)	0.01
C_6_H_8_O_7_ (25 mmol/L)	0.03
CaCl_2_ (15 mmol/L)	0.17
**Gastric juice (pH = 2)**	
NaCl	2.00
Pepsin	3.20
HCl	0.12
**Intestinal juice (pH = 8)**	
Pancreatic extract	0.02
Bile salt	0.12
NaHCO_3_	8.40

**Table 2:** The concentration of metals (Mg and Zn) and anions (Cl^−^ and SO_4_^2−^) extracted into the artificial digestive juices from the biomass of P.c—*Pleurotus citrinopileatus,* P.d*—Pleurotus djamor*, and P.p*—Pleurotus pulmonarius* (mg/100 d.w.).

Species	Sample	Addition	Mg (mg/100 g d.w.)		Zn (mg/100 g d.w.)		Cl^−^ (mg/100 g d.w.)		SO_4_^2−^ (mg/100 g d.w.)	
			Gastric Juice	Intestinal Juice	Gastric Juice	Intestinal Juice	Gastric Juice	Intestinal Juice	Gastric Juice	Intestinal Juice
**P.c**	Fruiting bodies		103.4 ± 9.2 ^b^	28.1 ± 1.6 ^b^	12.2 ± 1.2 ^a^	2.1 ± 0.4 ^a^	667 ± 5 ^b^	464 ± 2 ^c^	404 ± 0 ^a^	196 ± 1 ^b^
	Mycelium		51.2 ± 4.7 ^a^	20.2 ± 0.5 ^a^	10.5 ± 0.1 ^a^	1.1 ± 0.1 ^a^	160 ± 4 ^a^	78 ± 2 ^a^	416 ± 1 ^b^	93 ± 1 ^a^
	Mycelium	MgSO_4_·7H_2_O	906.2 ± 25.1 ^d^	88.4 ± 4.1 ^d^	-	-	-	-	1302 ± 3 ^c^	90 ± 6 ^a^
	Mycelium	MgCl_2_·6H_2_O	824.9 ± 17.7 ^c^	76.7 ± 2.0 ^c^	-	-	867 ± 9 ^c^	285 ± 3 ^b^	-	-
	Mycelium	ZnSO_4_·7H_2_O	-	-	166.3 ± 3.4 ^c^	11.7 ± 1.5 ^c^	-	-	408 ± 2 ^a^	199 ± 1 ^b^
	Mycelium	ZnCl_2_	-	-	111.3 ± 0.8 ^b^	6.4 ± 0.6 ^b^	1067 ± 8 ^d^	464 ± 6 ^c^	-	-
**P.d**	Fruiting bodies		71.1 ± 5.7 ^a^	8.2 ± 0.8 ^a^	10.3 ± 0.1 ^a^	1.8 ± 0.1 ^a^	200 ± 2 ^b^	70 ± 1 ^a^	226 ± 3 ^a^	121 ± 2 ^b^
	Mycelium		59.2 ± 4.7 ^a^	10.4 ± 1.3 ^a^	13.9 ± 0.3 ^a^	0.9 ± 0.1 ^a^	400 ± 2 ^c^	133 ± 1 ^b^	626 ± 1 ^b^	262 ± 1 ^d^
	Mycelium	MgSO_4_·7H_2_O	842.8 ± 8.5 ^c^	155.9 ± 5.8 ^c^	-	-	-	-	2462 ± 5 ^d^	91 ± 3 ^a^
	Mycelium	MgCl_2_·6H_2_O	697.8 ± 12.5 ^b^	117.0 ± 3.4 ^b^	-	-	667 ± 3 ^d^	305 ± 18 ^c^	-	-
	Mycelium	ZnSO_4_·7H_2_O	-	-	145.8 ± 6.7 ^c^	9.6 ± 1.7 ^b^	-	-	678 ± 1 ^c^	253 ± 1 ^c^
	Mycelium	ZnCl_2_	-	-	128.1 ± 4.2 ^b^	11.6 ± 2.3 ^b^	133 ± 8 ^a^	68 ± 13 ^a^	-	-
**P.p**	Fruiting bodies		59.1 ± 4.8 ^a^	10.4 ± 1.3 ^a^	6.9 ± 0.6 ^a^	1.4 ± 0.2 ^a^	600 ± 1 ^c^	410 ± 2 ^c^	287 ± 0 ^a^	21 ± 1 ^a^
	Mycelium		41.1 ± 4.2 ^a^	8.3 ± 0.7 ^a^	15.3 ± 0.7 ^a^	2.0 ± 0.3 ^a^	1000 ± 2 ^d^	413 ± 2 ^c^	449 ± 0 ^b^	68 ± 1 ^b^
	Mycelium	MgSO_4_·7H_2_O	1280.4 ± 16.5 ^c^	82.3 ± 3.9 ^b^	-	-	-	-	3636 ± 3 ^d^	399 ± 3 ^d^
	Mycelium	MgCl_2_·6H_2_O	526.9 ± 19.7 ^b^	131.6 ± 5.1 ^c^	-	-	400 ± 4 ^a^	133 ± 3 ^a^	-	-
	Mycelium	ZnSO_4_·7H_2_O	-	-	121.8 ± 3.6 ^c^	22.3 ± 0.2 ^c^	-	-	818 ± 2 ^c^	385 ± 1 ^c^
	Mycelium	ZnCl_2_	-	-	98.8 ± 5.5 ^b^	16.4 ± 1.1 ^b^	533 ± 5 ^b^	385 ± 3 ^b^	-	-

N = 9; dash (-) indicates not analyzed; values followed by a different letter (a, b, c, d) within the same row are significantly different (*p* < 0.05).

**Table 3:** Phenolic and indole compounds content extracted into the artificial digestive juices from the biomass obtained from in vitro cultures of P.c*— Pleurotus citrinopileatus*, P.d*—Pleurotus djamor*, and P.p*—Pleurotus pulmonarius* (mg/100 d.w.).

Species	Sample	Addition	Phenylalanine and Phenolic Acids [mg/100 g d.w.]									
			Phenylalanine		Gallic Acid		Protocatechuic Acid		*p*-Hydroxybenzoic Acid			
			Gastric Juice	Intestinal Juice	Gastric Juice	Intestinal Juice	Gastric Juice	Intestinal Juice	Gastric Juice	Intestinal Juice		
**P.c**	Fruiting bodies		108.3 ± 13.9 ^a^	n.d.	n.d	n.d	0.14 ± 0.01 ^a^	n.d.	0.14 ± 0.01 ^cd^	n.d.		
	Mycelium		395.2 ± 57.9 ^d^	n.d.	4.49 ± 0.75 ^d^	n.d	0.13 ± 0.02 ^a^	n.d.	0.13 ± 0.01 ^bc^	n.d.		
	Mycelium	MgSO_4_·7H_2_O	271.0 ± 1.7 ^c^	37.2 ± 1.9 ^d^	0.78 ± 0.01 ^b^	0.35 ± 0.01 ^b^	0.15 ± 0.02 ^a^	0.35 ± 0.01 ^b^	0.15 ± 0.01 ^d^	0.22 ± 0.01 ^c^		
	Mycelium	MgCl_2_·6H_2_O	168.7 ± 2.9 ^b^	24.2 ± 0.4 ^b^	0.25 ± 0.11 ^a^	n.d	n.d.	n.d.	0.07 ± 0.01 ^a^	0.02 ± 0.00 ^b^		
	Mycelium	ZnSO4·7H_2_O	448.0 ± 22.1 ^d^	26.7 ± 0.5 ^c^	2.43 ± 0.08 ^c^	0.15 ± 0.05 ^a^	n.d.	n.d.	0.12 ± 0.01 ^b^	0.01 ± 0.00 ^a^		
	Mycelium	ZnCl_2_	127.8 ± 6.1 ^ab^	12.8 ± 1.2 ^a^	n.d	0.12 ± 0.00 ^a^	n.d.	0.16 ± 0.01 ^b^	0.22 ± 0.01 ^e^	n.d.		
**P.d**	Fruiting bodies		24.9 ± 1.7 ^a^	n.d.	n.d.	n.d.	n.d.	0.03 ± 0.01 ^a^	0.03 ± 0.01 ^a^	n.d.		
	Mycelium		657.5 ± 13.5 ^e^	n.d.	2.10 ± 0.21 ^b^	n.d.	n.d.	n.d.	0.23 ± 0.01 ^d^	n.d.		
	Mycelium	MgSO_4_·7H_2_O	563.9 ± 1.6 ^d^	32.7 ± 0.9 ^a^	n.d.	n.d.	n.d.	n.d.	0.12 ± 0.03 ^c^	n.d.		
	Mycelium	MgCl_2_·6H_2_O	262.0 ± 8.7 ^b^	33.2 ± 10.4 ^a^	1.13 ± 0.31 ^a^	n.d.	n.d.	0.14 ± 0.02 ^b^	0.79 ± 0.01 ^e^	0.14 ± 0.00 ^b^		
	Mycelium	ZnSO_4_·7H_2_O	721.6 ± 1.1^f^	217.5 ± 32.7 ^b^	n.d.	n.d.	n.d.	0.03 ± 0.02 ^a^	0.06 ± 0.01 ^b^	0.03 ± 0.00 ^a^		
	Mycelium	ZnCl_2_	547.5 ± 1.1 ^c^	42.8 ± 0.5 ^a^	n.d.	n.d.	n.d.	n.d	0.23 ± 0.01 ^d^	n.d.		
**P.p**	Fruiting bodies		137.7 ± 3.3 ^b^	n.d.	n.d.	n.d.	n.d.	n.d.	n.d.	n.d.		
	Mycelium		49 ± 5.1 ^a^	n.d	0.26 ± 0.01 ^b^	n.d.	n.d.	n.d.	n.d.	n.d.		
	Mycelium	MgSO_4_·7H_2_O	274.4 ± 9.6 ^d^	23.8 ± 2.5 ^b^	0.15 ± 0.02 ^a^	n.d.	n.d.	n.d.	n.d.	n.d.		
	Mycelium	MgCl_2_·6H_2_O	35.7 ± 0.1 ^a^	3.7 ± 0.1 ^a^	n.d.	n.d.	n.d.	n.d.	n.d.	n.d.		
	Mycelium	ZnSO_4_·7H_2_O	320.1 ± 0.8 ^e^	55.3 ± 3.8 ^d^	n.d.	n.d.	n.d.	n.d.	n.d.	n.d.		
	Mycelium	ZnCl_2_	183.7 ± 34.3 ^c^	36.8 ± 1.7 ^c^	n.d.	n.d.	n.d.	n.d.	n.d.	n.d.		
**Species**	**Sample**	**Addition**	**Indole Compounds [mg/100 g d.w.]**									
			**5-Hydroxytryptophan**		**L-Tryptophan**		**Melatonin**		**Tryptamine**		**Serotonin**	
			**Gastric** **Juice**	**Intestinal** **Juice**	**Gastric** **Juice**	**Intestinal Juice**	**Gastric** **Juice**	**Intestinal** **Juice**	**Gastric** **Juice**	**Intestinal** **Juice**	**Gastric** **Juice**	**Intestinal** **Juice**
**P.c**	Fruiting bodies		0.47 ± 0.01 ^a^	0.17 ± 0.01 ^bc^	3.15 ± 0.01 ^a^	n.d.	n.d.	n.d.	n.d.	n.d.	n.d.	n.d.
	Mycelium		1.63 ± 0.07 ^d^	0.19 ± 0.01 ^c^	10.61 ± 0.50 ^c^	n.d.	n.d.	n.d.	n.d.	n.d.	n.d.	n.d.
	Mycelium	MgSO_4_·7H_2_O	0.77 ± 0.06 ^b^	0.13 ± 0.08	n.d.	2.87 ± 0.01 ^a^	*	*	n.d.	n.d.	n.d.	n.d.
	Mycelium	MgCl_2_·6H_2_O	0.39 ± 0.11 ^a^	0.11 ± 0.01 ^abc^	4.07 ± 0.10 ^b^	2.99 ± 0.01 ^b^	*	n.d.	*	n.d.	n.d.	n.d.
	Mycelium	ZnSO_4_·7H_2_O	1.06 ± 0.06 ^c^	0.05 ± 0.03 ^a^	n.d.	3.19 ± 0.01 ^c^	*	*	n.d.	n.d.	0.04 ± 0.0	n.d.
	Mycelium	ZnCl_2_	0.45 ± 0.02 ^a^	0.07 ± 0.04 ^ab^	3.74 ± 0.01 ^b^	3.69 ± 0.01 ^d^	*	*	n.d.	n.d.	n.d	n.d.
**P.d**	Fruiting bodies		0.29 ± 0.04 ^a^	n.d.	4.06 ± 0.01 ^d^	n.d.	n.d.	n.d.	n.d.	n.d.	n.d.	n.d.
	Mycelium		n.d.	n.d.	2.74 ± 0.01 ^a^	n.d.	n.d.	n.d.	n.d.	n.d.	n.d.	n.d.
	Mycelium	MgSO_4_·7H_2_OO	0.29 ± 0.01 ^a^	0.11 ± 0.01 ^a^	4.24 ± 0.03 ^e^	3.53 ± 0.01 ^b^	n.d.	n.d.	n.d.	n.d.	n.d.	n.d.
	Mycelium	MgCl_2_·6H_2_O	0.51 ± 0.01 ^b^	0.11 ± 0.00 ^a^	5.01 ± 0.01 ^f^	3.63 ± 0.01 ^b^	n.d.	n.d.	n.d.	n.d.	n.d.	n.d.
	Mycelium	ZnSO_4_·7H_2_O	0.83 ± 0.04 ^d^	0.29 ± 0.02 ^b^	3.33 ± 0.01 ^b^	3.31 ± 0.02 ^a^	*	n.d.	n.d.	n.d.	n.d.	n.d.
	Mycelium	ZnCl_2_	0.57 ± 0.01 ^c^	0.12 ± 0.01 ^a^	3.64 ± 0.16 ^c^	4.10 ± 0.15 ^c^	n.d.	n.d.	n.d.	n.d.	n.d.	n.d.
**P.p**	Fruiting bodies		0.02 ± 0.01 ^a^	n.d.	10.61 ± 0.62 ^d^	n.d.	n.d.	n.d.	n.d.	n.d.	n.d.	n.d.
	Mycelium		0.31 ± 0.15 ^b^	0.38 ± 0.02 ^d^	3.68 ± 0.02 ^a^	n.d.	n.d.	n.d.	n.d.	n.d.	n.d.	n.d.
	Mycelium	MgSO_4_·7H_2_O	0.41 ± 0.08 ^b^	0.13 ± 0.01 ^b^	6.41 ± 0.02 ^c^	4.60 ± 0.02 ^b^	n.d.	n.d.	n.d.	n.d.	n.d.	n.d.
	Mycelium	MgCl_2_·6H_2_O	0.30 ± 0.01 ^b^	0.05 ± 0.03 ^a^	4.72 ± 0.08 ^b^	3.78 ± 0.01 ^a^	n.d.	n.d.	n.d.	*	n.d.	n.d.
	Mycelium	ZnSO_4_·7H_2_O	0.93 ± 0.03 ^d^	0.22 ± 0.03 ^c^	3.24 ± 0.01 ^a^	5.33 ± 0.77 ^c^	n.d.	*	n.d.	n.d.	n.d.	n.d.
	Mycelium	ZnCl_2_	0.60 ± 0.05 ^d^	0.15 ± 0.01 ^c^	5.02 ± 0.01 ^a^	4.54 ± 0.05 ^c^	n.d.	n.d.	n.d.	n.d.	n.d.	n.d.

N = 9; n.d.—not detected; *—lower than the limit of detection, values followed by a different letter (a, b, c, d, e, f) within the same row are significantly different (*p* < 0.05).

**Table 4:** Values of loadings of three components PC1, PC2, and PC3.

	PC1	PC2	PC3
Phenylalanine	0.46508	−0.49099	−0.07265
Gallic acid	0.53583	0.04218	0.32777
Protocatechuic acid	0.23264	0.83861	0.16821
*p*-Hydroxybenzoic acid	0.22460	0.19685	−0.58975
L-Tryptophan	−0.23189	−0.08289	0.68890
5-Hydroxytryptophan	0.58157	−0.09077	0.19126

**Table 5:** Total phenol content and antioxidant activity (percentage of DPPH reduction) in dry material, artificial gastric juice, and artificial intestinal juice of P.c*—Pleurotus citrinopileatus*, P.d*—Pleurotus djamor*, and P.p*—Pleurotus pulmonarius* (mg/100 d.w.).

Species	Sample	Addition	TP	DPPH	TP	DPPH	TP	DPPH
			Dry Material	Dry Material	Gastric Juice	Gastric Juice	Intestinal Juice	Intestinal Juice
**P.c**	Fruiting bodies		297 ± 24 ^e^	44 ± 0.5 ^d^	4.33 ± 0.03 ^b^	8.9 ± 0.41 ^b^	1.3 ± 0.01 ^c^	1.18 ± 0.21 ^c^
	Mycelium		243 ± 6 ^d^	14.4 ± 0.2 ^c^	8.39 ± 0.12 ^f^	33.86 ± 0.22 ^f^	2.28 ± 0.01 ^e^	0.51 ± 0.14 ^ab^
	Mycelium	MgSO_4_·7H_2_O	98 ± 3 ^a^	7 ± 0.2 ^a^	5.77 ± 0.01 ^d^	20.81 ± 0.55 ^d^	1.07 ± 0.02 ^b^	0.54 ± 0.06 ^ab^
	Mycelium	MgCl_2_·6H_2_O	229 ± 2 ^d^	12.9 ± 0.6 ^b^	4.49 ± 0.03 ^c^	10.82 ± 0.68 ^c^	1.09 ± 0.02 ^b^	2.38 ± 0.17 ^d^
	Mycelium	ZnSO4·7H_2_O	135 ± 1 ^b^	4.7 ± 0.2 ^a^	6.91 ± 0.02 ^e^	31.84 ± 0.07 ^e^	1.4 ± 0.03 ^d^	0.85 ± 0.01 ^bc^
	Mycelium	ZnCl_2_	192 ± 12 ^c^	5.6 ± 0.8 ^a^	4.13 ± 0.01 ^a^	7.25 ± 0.22 ^a^	0.13 ± 0.01 ^a^	0.25 ± 0.09 ^a^
**P.d**	Fruiting bodies		161 ± 1 ^c^	10.3 ± 0.4 ^b^	3.73 ± 0.03 ^a^	5.51 ± 0.36 ^a^	1.8 ± 0.01 ^d^	9.15 ± 0.14 ^d^
	Mycelium		193 ± 8 ^d^	11.5 ± 0.5 ^b^	7.67 ± 0.01 ^f^	25.16 ± 0.43 ^e^	2.2 ± 0.02 ^f^	10.11 ± 1.15 ^d^
	Mycelium	MgSO_4_·7H_2_O	105 ± 6 ^a^	6 ± 0.2 ^a^	4.41 ± 0.06 ^b^	9.52 ± 0.21 ^b^	0.88 ± 0.01 ^a^	2.04 ± 0.4 ^c^
	Mycelium	MgCl_2_·6H_2_O	186 ± 3 ^d^	11.3 ± 2.2 ^b^	5.18 ± 0.02 ^c^	10.66 ± 0.36 ^c^	1.35 ± 0.01 ^c^	1.01 ± 0.16 ^b^
	Mycelium	ZnSO_4_·7H_2_O	142 ± 3 ^b^	9 ± 1.1 ^b^	6.22 ± 0.02 ^e^	20.34 ± 0.41 ^d^	1.95 ± 0.01 ^e^	0.29 ± 0.01 ^a^
	Mycelium	ZnCl_2_	230 ± 1 ^e^	9.5 ± 0.3 ^b^	5.83 ± 0.01 ^d^	9.93 ± 0.08 ^bc^	1.01 ± 0.02 ^b^	0.29 ± 0.01 ^a^
**P.p**	Fruiting bodies		92 ± 13 ^a^	7.8 ± 0.5 ^ab^	8.34 ± 0.03 ^d^	43.43 ± 0.07 ^f^	2.13 ± 0.01 ^e^	5.82 ± 0.07 ^d^
	Mycelium		259 ± 5 ^c^	19 ± 0.7 ^e^	4.88 ± 0.01 ^b^	4.38 ± 0.66 ^b^	2.32 ± 0.01 ^f^	3.2 ± 0.36 ^c^
	Mycelium	MgSO_4_·7H_2_O	141 ± 3 ^b^	3.9 ± 0.3 ^a^	4.73 ± 0.17 ^b^	7.27 ± 0.43 ^c^	0.97 ± 0.01 ^b^	5.16 ± 0.39 ^d^
	Mycelium	MgCl_2_·6H_2_O	253 ± 5 ^c^	14 ± 0.7 ^c^	3.89 ± 0.03 ^a^	3.22 ± 0.21 ^a^	0.91 ± 0.01 ^a^	1.24 ± 0.3 ^b^
	Mycelium	ZnSO_4_·7H_2_O	125 ± 0 ^b^	10 ± 0.2 ^bc^	6.33 ± 0.06 ^c^	18.36 ± 0.22 ^e^	1.71 ± 0.01 ^d^	3.35 ± 0.07 ^c^
	Mycelium	ZnCl_2_	104 ± 2 ^a^	3.9 ± 0.2 ^a^	6.45 ± 0.04 ^c^	14.01 ± 0.13 ^d^	1.49 ± 0.02 ^c^	0.29 ± 0.01 ^a^

N = 9; DPPH—1,1-diphenyl-2-picrylhydrazyl; TP—total phenols; values followed by a different letter (a, b, c, d, e) within the same row are significantly different (*p* < 0.05).

## Data Availability

Data is contained within the article.

## Samples Availability

Samples of the compounds are not available from the authors.
